# mTORC1 inhibition impairs activation of the unfolded protein response and induces cell death during ER stress in cardiomyocytes

**DOI:** 10.1152/ajpheart.00682.2022

**Published:** 2023-06-09

**Authors:** Christoph Hofmann, Zoe Löwenthal, Marjan Aghajani, Randal J. Kaufman, Hugo A. Katus, Norbert Frey, Christopher C. Glembotski, Mirko Völkers, Shirin Doroudgar

**Affiliations:** ^1^Division of Cardiology, Angiology, and Pneumology, Department of Internal Medicine III, Heidelberg University Hospital, Heidelberg, Germany; ^2^German Center for Cardiovascular Research (DZHK), Partner Site Heidelberg/Mannheim, Heidelberg, Germany; ^3^SDSU Heart Institute and Department of Biology, San Diego State University, San Diego, California, United States; ^4^Faculty of Biosciences, Heidelberg University, Heidelberg, Germany; ^5^Department of Internal Medicine and the Translational Cardiovascular Research Center, University of Arizona College of Medicine–Phoenix, Phoenix, Arizona, United States; ^6^Degenerative Diseases Program, Center for Genetic Disorders and Aging Research, Sanford Burnham Prebys Medical Discovery Institute, La Jolla, California, United States

**Keywords:** ATF6, cardiomyocytes, cell death, ER stress, mTORC1

## Abstract

The mechanistic target of rapamycin complex 1 (mTORC1) is a central regulator of protein synthesis that senses and responds to a variety of stimuli to coordinate cellular metabolism with environmental conditions. To ensure that protein synthesis is inhibited during unfavorable conditions, translation is directly coupled to the sensing of cellular protein homeostasis. Thus, translation is attenuated during endoplasmic reticulum (ER) stress by direct inhibition of the mTORC1 pathway. However, residual mTORC1 activity is maintained during prolonged ER stress, which is thought to be involved in translational reprogramming and adaption to ER stress. By analyzing the dynamics of mTORC1 regulation during ER stress, we unexpectedly found that mTORC1 is transiently activated in cardiomyocytes within minutes at the onset of ER stress before being inhibited during chronic ER stress. This dynamic regulation of mTORC1 appears to be mediated, at least in part, by ATF6, as its activation was sufficient to induce the biphasic control of mTORC1. We further showed that protein synthesis remains dependent on mTORC1 throughout the ER stress response and that mTORC1 activity is essential for posttranscriptional induction of several unfolded protein response genes. Pharmacological inhibition of mTORC1 increased cell death during ER stress, indicating that the mTORC1 pathway serves adaptive functions during ER stress in cardiomyocytes potentially by controlling the expression of protective unfolded protein response genes.

**NEW & NOTEWORTHY** Cells coordinate translation rates with protein quality control to ensure that protein synthesis is initiated primarily when proper protein folding can be achieved. Long-term activity of the unfolded protein response is therefore associated with an inhibition of mTORC1, a central regulator of protein synthesis. Here, we found that mTORC1 is transiently activated early in response to ER stress before it is inhibited. Importantly, partial mTORC1 activity remained essential for the upregulation of adaptive unfolded protein response genes and cell survival in response to ER stress. Our data reveal a complex regulation of mTORC1 during ER stress and its involvement in the adaptive unfolded protein response.

## INTRODUCTION

Protein synthesis requires a significant amount of cellular energy resources (1) and, as such, is tightly regulated in response to proteotoxic stresses, such as endoplasmic reticulum (ER) stress (2). To reduce the protein load on the ER and to prevent protein misfolding, the ER stress response attenuates translation, which is mediated by phosphorylation of the α-subunit of eukaryotic translation initiation factor 2 (eIF2α) and the inhibition of the mechanistic target of rapamycin complex 1 (mTORC1) pathway, both of which are key regulators of protein synthesis (3–5). Although PERK-dependent phosphorylation of eIF2α mediates an acute suppression of translation, resulting in attenuated protein load on the ER, ATF4 expression during prolonged ER stress again increases translation rates by GADD34-dependent dephosphorylation of eIF2α and partial activation of the mTORC1 pathway (4). However, recent evidence suggests that the interplay between ER stress and the mTORC1 pathway is more complex than previously anticipated, indicating that mTORC1 may not only act downstream of the ER stress response pathway but may itself also serve as an important regulator of ER stress response signaling (6–8). Although it has recently been proposed that such an interplay controls cardiomyocyte fate under conditions of increased protein processing (7), details of the cross talk between ER stress signaling and the mTORC1 pathway have not been fully elucidated.

In mammalian cells, the ER stress response or the unfolded protein response (UPR) is controlled by several ER transmembrane receptors of protein misfolding, including activating transcription factor 6α (ATF6) (5). Exceeding the folding capacity of the ER results in activation of ATF6, which balances protein synthesis and folding to maintain cellular protein homeostasis. Unexpectedly, ATF6 was previously shown to possess intrinsic capability to activate the mTORC1 pathway under certain conditions, which was described as noncanonical ATF6 signaling (6, 7, 9, 10). Yet, how the mTORC1 pathway is regulated by ATF6 in response to ER stress and how this may contribute to the maintenance of cellular homeostasis remain incompletely understood.

By analyzing the dynamic cellular response to ER stress, we found that mTORC1 is transiently activated within minutes of the onset of disruption of ER protein homeostasis, followed by incomplete inhibition of mTORC1 activity during prolonged ER stress. We further demonstrate that this biphasic regulation of the mTORC1 pathway can be mediated by ATF6 and that pharmacological inhibition of residual mTORC1 activity during prolonged ER stress impairs the posttranscriptional activation of the UPR, resulting in increased cell death in cardiomyocytes.

## METHODS

### Cell Culture and Chemicals

Neonatal rat ventricular cardiomyocytes (NRCMs) were isolated as previously described (11) and plated at a density of 0.5 × 10^6^ cells per well on six-well (34.8 mm) plastic plates that had been pretreated with 5 μg/mL fibronectin in serum-free DMEM/F12 (1:1) medium for 1 h and then cultured in DMEM-F12, containing 10% fetal bovine serum (FBS), 100 U/mL penicillin, 100 µg/mL streptomycin, and 292 μg/mL glutamine. On the next day, media was changed to 2 mL DMEM-F12 medium containing 2% FBS, penicillin, streptomycin, and glutamine. After 24 h, cultures were subjected to the respective treatments.

Immortalized WT and ATF6 knockout mouse embryonic fibroblasts (MEFs) were a kind gift from Randal Kaufman (12). MEFs were seeded at a density of 0.3 × 10^6^ cells per well on noncoated six-well plastic plates and cultured similarly to NRCMs except that cells were cultured in 2 mL of DMEM medium. Cultures were treated with 10 μg/mL tunicamycin (TM; Cat. No. T7765, Merck), 1 mM dithiothreitol (DTT; Cat. No. 43819, Sigma-Aldrich), 100 nM rapamycin (Cat. No. sc-3504B, Santa Cruz Biotechnology), 100 nM Torin1 (Cat. No. Cay-10997, Cayman Chemical), or 10 µM compound 147. Compound 147 was a kind gift from Luke Wiseman. Respective treatment times can be found in the figure legends.

### Puromycin Incorporation Assay

Changes in protein synthesis were measured by puromycin incorporation. Puromycin (0.5 μg/mL) was added to the culture medium 30 min before harvesting the cells. Thereafter, cells were washed once with ice-cold phosphate-buffered saline (PBS) and then harvested for immunoblotting.

### Immunoblotting

Samples usually comprising 5–20 µg of protein were mixed with Laemmli buffer containing 2-mercaptoethanol, heated for 10 min at 75°C, separated by SDS-PAGE, and transferred to a PVDF membrane. The following antibodies were used to probe the membranes: puromycin 1:5,000 (Cat. No. MABE343, Merck Millipore), mTOR 1:1,000 (Cat. No. 2983, Cell Signaling Technology), raptor 1:1,000 (Cat. No. 2280, Cell Signaling Technology), phosphorylated p70 S6 kinase Thr389 1:1,000 (Cat. No. 9205, Cell Signaling Technology), phosphorylated ribosomal S6 Ser235/236 1:5,000 (Cat. No. 4858, D57.2.2E, Cell Signaling Technology), ribosomal S6 1:1,000 (Cat. No. 2317, 54D2, Cell Signaling Technology), phosphorylated 4EBP1 Thr37/46 1:5,000 (Cat. No. 2855, 236B4, Cell Signaling Technology), 4EBP1 1:5,000 (Cat. No. 9452, Cell Signaling Technology), phosphorylated ERK1/2 Thr202/Tyr204 1:1,000 (Cat. No. 9101, Cell Signaling Technology), phosphorylated p38 MAPK Thr180/Tyr182 1:1,000 (Cat. No. 4511, D3F9, Cell Signaling Technology), phosphorylated MAPKAPK-2 Thr334 1:1,000 (Cat. No. 3007, 27B7, Cell Signaling Technology), phosphorylated Hsp27 Ser82 1:1,000 (Cat. No. 9709, D1H2F6, Cell Signaling Technology), phosphorylated JNK Thr183/Tyr185 1:1,000 (Cat. No. 4668, 81E11, Cell Signaling Technology), ATF6 1:1,000 (Cat. No. 24169-1-AP, Proteintec), KDEL 1:5,000 (used to detect GRP94, GRP78, and PDIA6) (Cat. No. ADI-SPA-827, Enzo Life Sciences), GAPDH 1:20,000 (Cat. No. G-9, sc-365062, Santa Cruz Biotechnology), and α-tubulin 1:10,000 (Cat. No. 2144, Cell Signaling Technology). Chemiluminescence was detected using X-ray film (Cat. No. 34091, Pierce) and either a Cawomat 2000IR or a Konica Minolta SRX-101A film processor following Western Lighting Plus-ECL Enhanced Chemiluminescence Substrate (Cat. No. NEL10400, Perkin Elmer) application. Densitometry was performed on Western blot bands to estimate protein quantity using ImageJ software. 

### Quantitative Real-Time PCR

Total RNA was isolated from cultured cardiomyocytes using the Quick-RNA MiniPrep Kit (Cat. No. R1055, Zymo Research) or TRIzol Reagent (Cat. No. 15596026, Invitrogen), according to the manufacturer’s instructions. cDNA was generated by reverse transcription using Superscript III First-Strand Synthesis System (Cat. No. 18080-051, Invitrogen). Quantitative real-time PCR was performed with Maxima SYBR Green/ROX qPCR Master Mix (Cat. No. K0222, Thermo Fisher) in a StepOnePlus RT-PCR System (Thermo Fisher). The following primers were used.

Rat Hsp90b1:

 Forward: AAGCATCTGATTACCTTGAATTGGAT

 Reverse: CTCCTCCACAGTCTCAGTCTTGCT

Rat Hprt:

 Forward: TCA GACCGCTTTTCCCGCGA

 Reverse: TCACTAATCACGACGCTGGGACTGAG

Rat and Mouse 18S:

 Forward: CGAGCCGCCTGGATACC

 Reverse: CATGGCCTCACTTCCGAAAA

Rat and Mouse Gapdh:

 Forward: GACAAACGTCTTCGGGAATTG

 Reverse: CTCTTTAAGGGCAGGGAGTATC

Rat and Mouse Hspa5:

 Forward: CACGTCCAACCCGGAGAA

 Reverse: GTAGCCTGCGTGAACCTTA

Mouse Atf6:

 Forward: CTTCCTCCAGTTGCTCCATC

 Reverse: CAACTCCTCAGGAACGTGCT

Mouse Manf:

 Forward: TGGGTGCGTTCTTCGACAT

 Reverse: GACGGTTGCTGGATCATTGAT

### Cell Number Quantification

Cells were washed twice with PBS to remove nonviable cells. Cells were then brought into suspension by treating cells with trypsin for ∼5 min until all cells were nonadherent. Digestion was stopped by adding DMEM-F12 medium containing 10% FBS. The cell suspension was transferred into a 15-mL falcon tube and centrifuged at 500 *g* for 5 min, after which the media was aspirated and the pellet was resuspended in 100 μL PBS. Cell concentration for each sample was then determined using a Scepter cell counter with a 60-μm sensor.

### Flow Cytometry–Apoptosis Assay

NRCMs were pretreated with 100 nM rapamycin or 100 nM Torin1 for 1 h, followed by 10 μg/mL TM treatment. Apoptosis was quantified after 48 h, using the Dead Cell Apoptosis Kits with Annexin V for Flow Cytometry (Cat. No. V13242, Invitrogen), according to the manufacturer’s protocol. Flow cytometry was performed using a BD FACSVerse flow cytometer.

### Statistical Analysis

Statistical analysis was performed using GraphPad Prism 7.0 (GraphPad Software). Data values are means ± SD. For statistical analysis, one-way ANOVA with Turkey post hoc analysis was used. *P* ≤ 0.05 was defined as significant difference. Biological replicate numbers for each figure can be found in the accompanying figure legend.

## RESULTS

**Figure 1.**
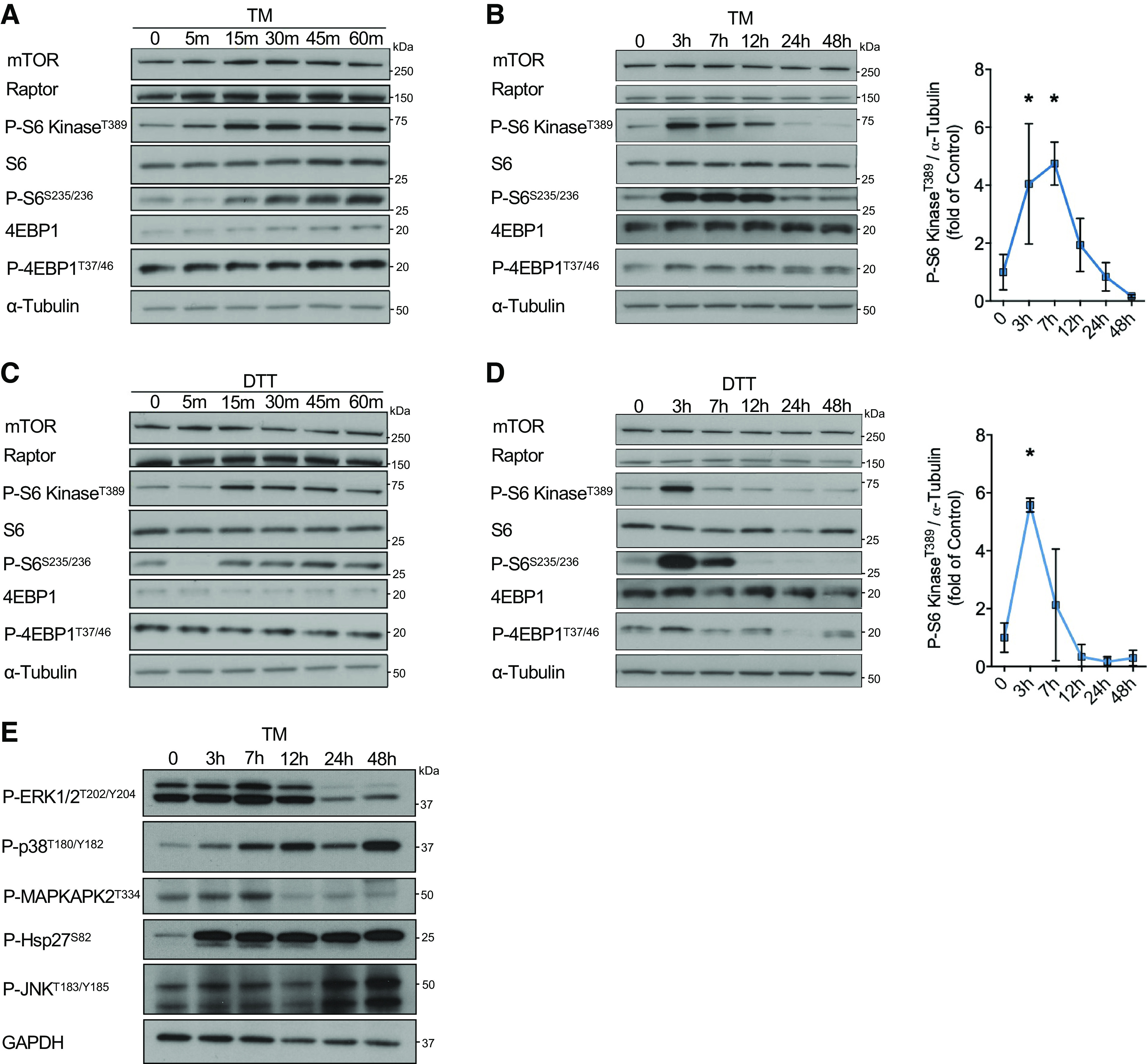
Transient activation of the mTORC1 pathway during the early phase of ER stress. *A*: immunoblot of mTORC1 signaling in NRCMs treated with 10 µg/mL TM for timepoints up to 1 h. *B*: immunoblot with quantification of mTORC1 signaling in NRCMs treated with 10 µg/mL TM for timepoints up to 48 h, *n* = 3. *C*: immunoblot of mTORC1 signaling in NRCMs treated with 1 mM DTT for timepoints up to 1 h. *D*: immunoblot with quantification of mTORC1 signaling in NRCMs treated with 1 mM DTT for timepoints up to 48 h, *n* = 3. *E*: immunoblot of several major signaling pathways regulated by ER stress in NRCMs treated with 10 µg/mL TM for timepoints up to 48 h. *P ≤ 0.05 from time-matched control in *A* or control in *C* and *E*. DTT, dithiothreitol; ER, endoplasmic reticulum; mTORC1, mechanistic target of rapamycin complex 1; NRCM, neonatal rat ventricular cardiomyocyte; TM, tunicamycin.

It is well established that ER stress results in the inhibition of mRNA translation via phosphorylation of the translation initiation factor eIF2 α-subunit (eIF2α), which reduces further protein load on the ER and enables active translation of specific mRNAs to maintain proteostasis (4). In general, this acute inhibition of translation is followed by a partial recovery of protein synthesis mediated by GADD34-dependent dephosphorylation of eIF2α (13). However, protein synthesis remains chronically suppressed during unrelieved ER stress because of the inhibition of the mTORC1 pathway. Yet, some mTORC1 activity is maintained, which is thought to be involved in translational reprogramming upon ER stress, but its function remains incompletely elucidated (4). To systematically analyze the regulation of the mTORC1 pathway during ER stress, we induced ER protein misfolding using the protein *N*-linked glycosylation inhibitor tunicamycin (TM) and quantified the phosphorylation of different downstream targets of mTORC1 implicated in protein synthesis (p70 S6 kinase, ribosomal protein S6, and the eukaryotic translation initiation factor 4E-binding protein 1, also known as 4EBP1) in isolated neonatal rat cardiomyocytes (NRCMs). Surprisingly, TM-induced ER stress resulted in immediate activation of the mTORC1 pathway in NRCMs within minutes, as indicated by increased phosphorylation levels of the examined downstream targets of mTORC1, which appeared to primarily involve the phosphorylation of S6 kinase and its downstream target S6 (Fig. 1*A*). Further analysis of mTORC1 activity at later timepoints after TM treatment revealed that the initial activation of mTORC1 for several hours, is followed by a second phase during chronic ER stress in which mTORC1 activity is suppressed (Fig. 1*B*). To confirm that this observation was not a response particular only to inhibition of glycosylation, but rather a general response to misfolded proteins, we treated NRCMs with dithiothreitol (DTT), a potent reducing agent that blocks disulfide bond formation between cysteine residues of proteins and induces ER stress within minutes of treatment. Similar to the findings we observed after TM treatment, DTT resulted in a biphasic regulation of the mTORC1 pathway and S6 kinase phosphorylation in cardiomyocytes (Fig. 1, *C* and *D*). This biphasic regulation appears to be a specific response of the ER stress signaling cascade to mTORC1, as other major signaling pathways affected by ER stress, such as those regulated by p38, ERK1/2, or JNK, did not exhibit such a biphasic activity pattern (Fig. 1*E*).

**Figure 2.**
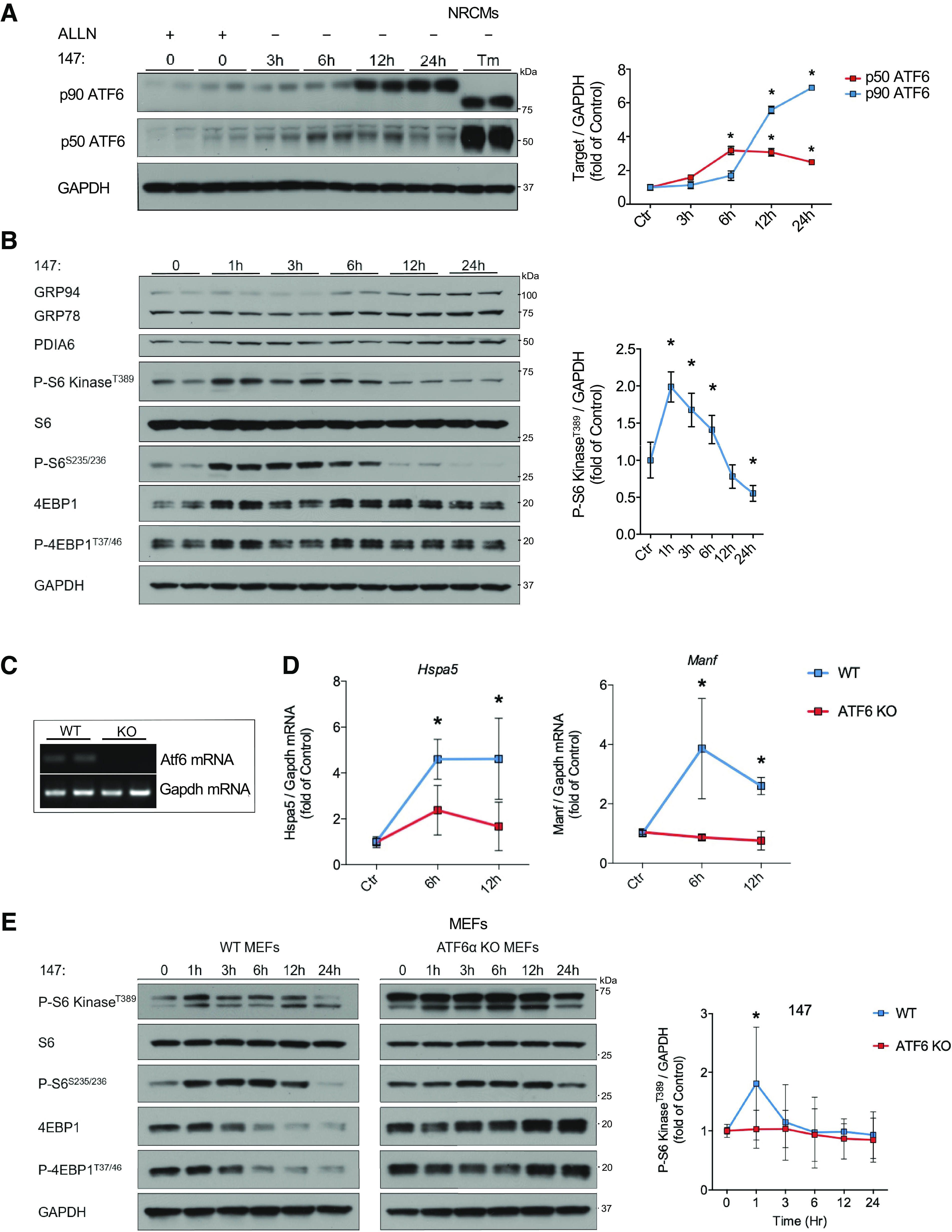
ATF6 is sufficient to transiently activate the mTORC1 pathway. *A*: p90 (full length) and p50 (active) ATF6 immunoblots and respective quantification in NRCMs treated with 10 µM 147 for increasing timepoints up to 24 h and 25 μg/mL ALLN (MG-101) 3 h before cell lysis. 147 treatment was initiated at different timepoints so that all cells were lysed at the same time. A positive control was treated with 10 µg/mL TM for 48 h. The two visible bands of p90 ATF6 represent its glycosylated and nonglycosylated forms after treatment with the glycosylation inhibitor TM, *n* = 2. *B*: immunoblot with quantification of mTORC1 signaling in NRCMs treated with 10 µM 147 for increasing timepoints up to 24 h, *n* = 6. *C*: RT-PCR of ATF6 to confirm full ATF6 KO in MEFs. *D*: mRNA levels of Hspa5 and Manf in WT and ATF6 KO MEFs 6 h and 12 h after 10 µM 147 treatment compared with untreated cells measured by RT-qPCR, *n* = 8–9. *E*: immunoblot and quantification of mTORC1 signaling in WT and ATF6 KO MEFs treated with 10 µM 147 for timepoints up to 24 h, *n* = 5. *P ≤ 0.05 from control. KO, knockout; mTORC1, mechanistic target of rapamycin complex 1; MEF, mouse embryonic fibroblast; NRCM, neonatal rat ventricular cardiomyocyte; TM, tunicamycin; WT, wild type.

ATF6 has been previously shown to possess the intrinsic ability to activate the mTORC1 pathway under certain conditions (7, 9). Therefore, we sought to determine whether the activation of the mTORC1 pathway during the acute phase of ER stress is mediated, at least in part, by ATF6 in cardiomyocytes. We used the small molecule *N*-(2-hydroxy-5-methylphenyl)-3-phenylpropanamide, also known as compound 147, to activate the ATF6 signaling axis of the ER stress response (Fig. 2*A*). The mechanism of action of compound 147 was recently described by Paxman et al. (14). Compound 147 is a prodrug that requires metabolic oxidation to form an electrophile which preferentially reacts with ER proteins to regulate ATF6 activity (14). Treatment with 147 resulted in the upregulation of GRP94, GRP78, and PDIA6, whose expression is controlled by ATF6 (15) (Fig. 2*B*). Similar to treatment with TM and DTT, 147 resulted in a biphasic regulation of the mTORC1/S6 kinase pathway in NRCMs with strong activation within the first hours after ATF6 activation, followed by mTORC1/S6 kinase suppression in response to chronic 147 treatment (Fig. 2*B*). Although 147 preferentially activates ATF6, it has also been shown to possess low activity toward the IRE1 branch of the ER stress response (15). To confirm that the regulation of mTORC1/S6 kinase by 147 is directly mediated by ATF6 and not derived from effects toward other signaling pathways, we treated WT and ATF6 KO mouse embryonic fibroblast (MEFs) with 147. ATF6 KO in MEFs was confirmed by RT-PCR (Fig. 2*C*). ATF6 KO MEFs showed no significant upregulation of the ATF6 target genes Hspa5 (GRP78) and Manf after treatment with 147 (Fig. 2*D*), confirming the high specificity of 147 toward ATF6 (15). Whereas WT MEFs showed similar regulation of mTORC1/S6 kinase as cardiomyocytes after 147 treatment (Fig. 2*E*), ATF6 KO MEFs lost their regulation of mTORC1/S6 kinase signaling in response to 147 treatment (Fig. 2*E*), confirming that the biphasic control of mTORC1/S6 kinase signaling can be directly mediated by ATF6.

**Figure 3.**
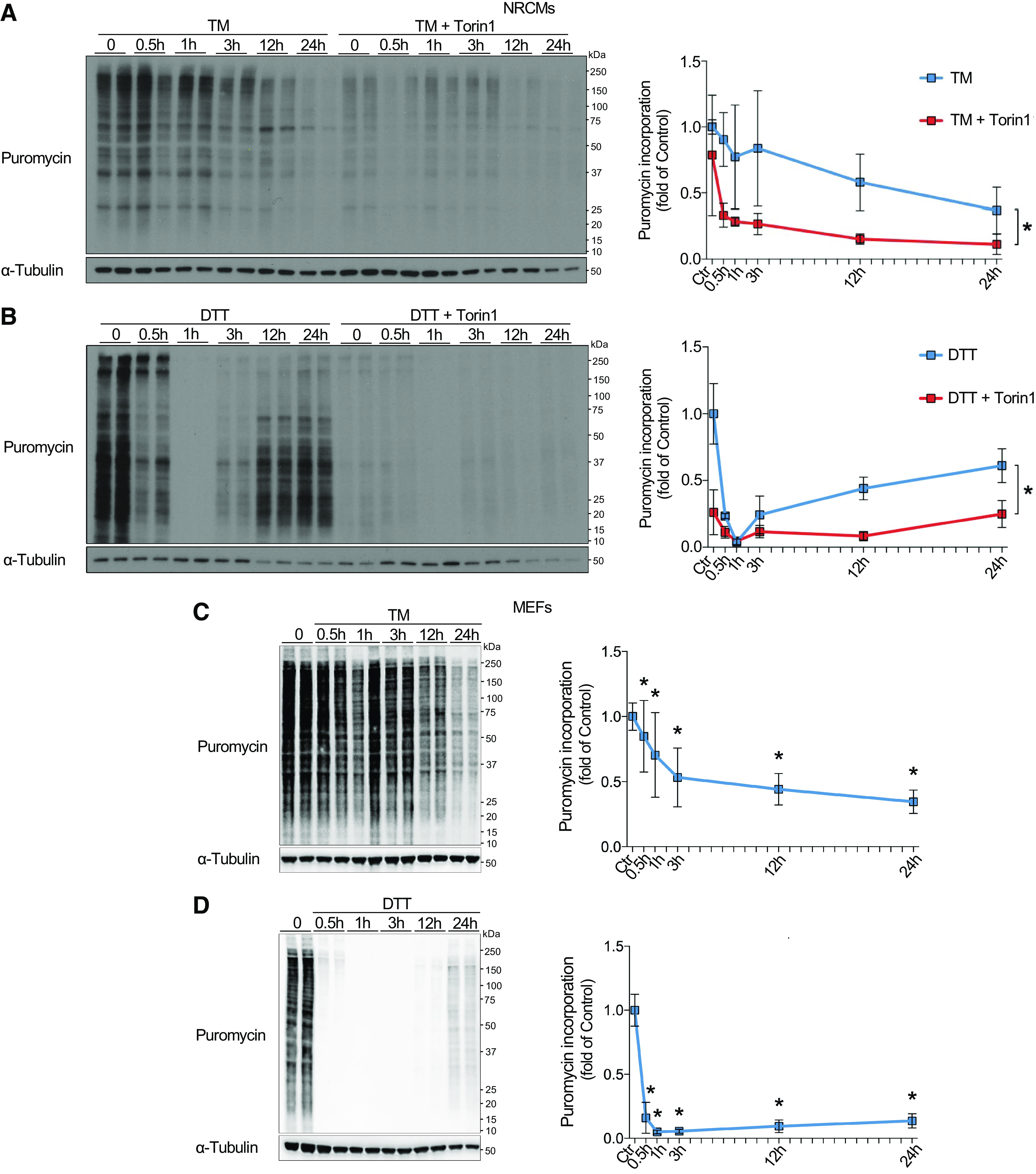
mTORC1-dependent regulation of translation during ER stress. *A* and *B*: puromycin immunoblots with quantification of NRCMs at increasing timepoints after 10 µg/mL TM (*A*) or 1 mM DTT (*B*) treatment. Cells were either pretreated with DMSO (vehicle, left) or 100 nM Torin1 (*right*) for 1 h, *n* = 6. *C* and *D*: puromycin immunoblots with quantification of MEFs at increasing timepoints after 10 µg/mL TM (*C*) or 1 mM DTT (*D*) treatment, *n* = 5. *P ≤ 0.05 from respective control. DTT, dithiothreitol; ER, endoplasmic reticulum; mTORC1, mechanistic target of rapamycin complex 1; NRCM, neonatal rat ventricular cardiomyocyte; TM, tunicamycin.

Previously, protein synthesis was reported to be regulated in the opposite manner to the changes in mTORC1 activity observed in this study, with acute suppression of translation, followed by partial recovery of translation rates at later phases of ER stress (16). To determine the impact of mTORC1 on translation during ER stress, NRCM protein synthesis rates were quantified using puromycin incorporation at different time points of ER stress induced by TM (Fig. 3*A*) or DTT (Fig. 3*B*) with or without pharmacological mTORC1 inhibition using the selective ATP-competitive mTORC1 inhibitor Torin1. Interestingly, differences in translational regulation were observed in response to TM and DTT treatment, with TM resulting in continuous downregulation of protein synthesis with increasing duration of treatment (Fig. 3*A*), whereas DTT resulted in acute inhibition of protein synthesis, followed by partial recovery (Fig. 3*B*). This indicates that mTORC1 activation during the early phase of ER stress does not necessarily result in increased global protein synthesis, potentially because of eIF2α phosphorylation-dependent inhibition of translation initiation. However, in both treatments, translation remained highly sensitive to mTORC1 inhibition by Torin1 (Fig. 3, *A* and *B*), suggesting that protein synthesis is at least partly dependent on residual mTORC1 activity during the acute and the chronic phase of ER stress in NRCMs. Because of previous reports of short-term inhibition of translation followed by partial recovery of protein synthesis during ER stress in MEFs (4), we also quantified puromycin incorporation in response to TM or DTT treatment in MEFs. The protein synthesis response of MEFs to ER stress was regulated in similar dynamics compared with NRCMs, indicating conserved mechanisms of translational regulation between cell types (Fig. 3, *C* and *D*).

**Figure 4.**
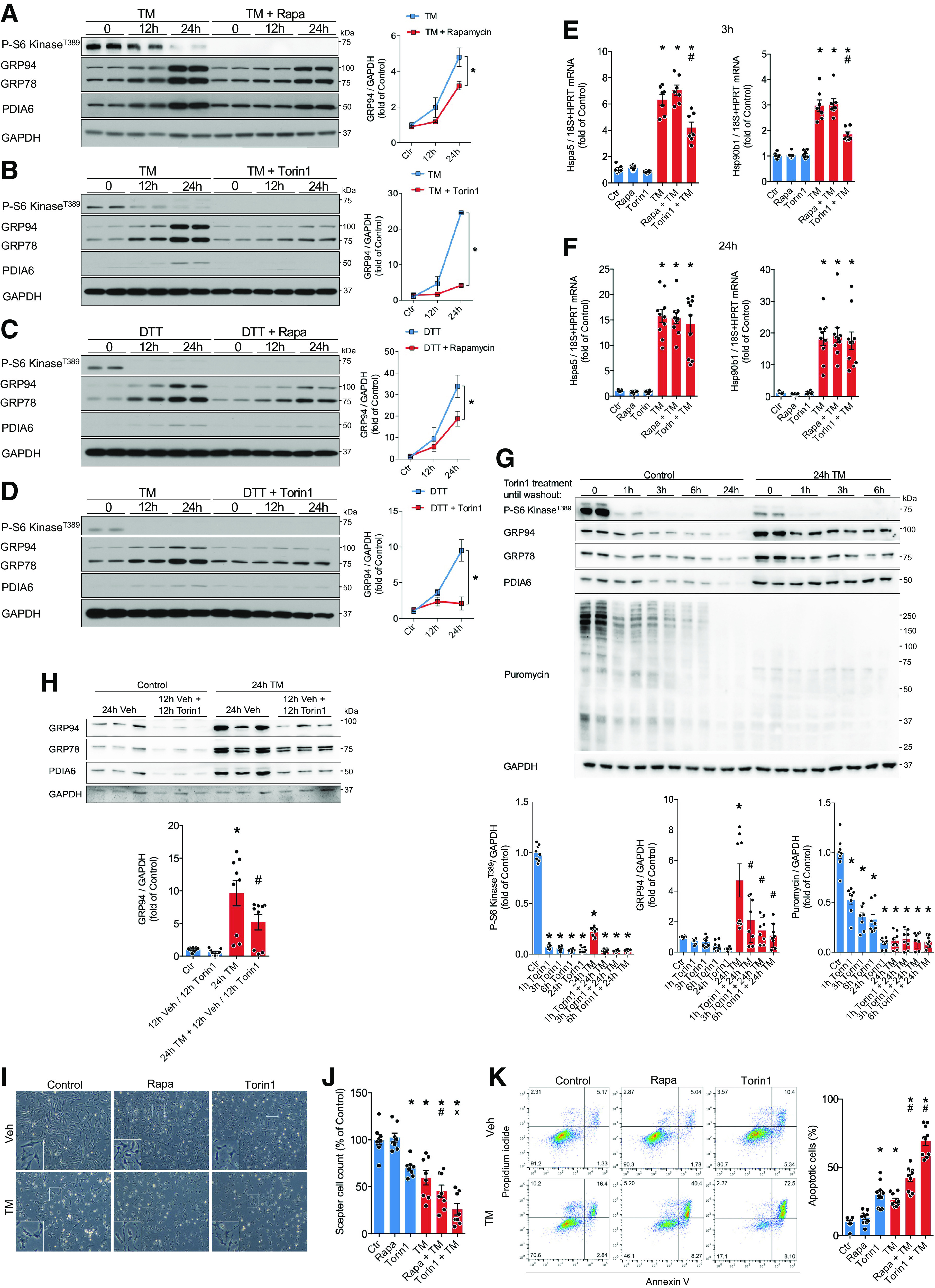
mTORC1 inhibition increases cell death in response to ER stress. *A* to *D*: immunoblots of P-S6 kinase^T389^ and ER proteostasis regulators and respective quantification in NRCMs 12 h and 24 h after 10 µg/mL TM (*A* and *B*) or 1 mM DTT (*C* and *D*) treatment. Cells were pretreated with 100 nM rapamycin (*A* and *C*) or 100 nM Torin1 for 1 h (*B* and *D*), *n* = 2. *E* and *F*: mRNA levels of Hsp90b1 and Hspa5 in NRCMs treated for 3 h (*E*) or 24 h (*F*) with 10 µg/mL TM and 100 nM rapamycin or 100 nM Torin1, *n* = 7–11. *G*: immunoblots of P-S6 kinase^T389^, ER proteostasis regulators, puromycin and respective quantification in NRCMs after 10 µg/mL TM and 100 nM Torin1 treatment for indicated timepoints, followed by washing with media and changing to new media containing only 10 µg/mL TM. Cells were lysed after 24 h total treatment, *n* = 8. *H*: immunoblots of P-S6 kinase^T389^ and ER proteostasis regulators and respective quantification in NRCMs after 12 h of 10 µg/mL TM treatment followed by 12 h of 10 µg/mL TM and 100 nM Torin1 treatment, *n* = 9. *I*: representative images of NRCMs 48 h after respective treatments. *J*: relative Scepter cell count 48 h after respective treatments, normalized to control, *n* = 8. *K*: quantification of NRCM apoptosis by FACS analysis 48 h after 10 µg/mL TM treatment and mTORC1 inhibition, *n* = 10. Representative FACS plots of each condition are shown on the left. *P ≤ 0.05 from time-matched control. #*P* ≤ 0.05 from TM in *E* and *G*, and *P* ≤ 0.05 from rapamycin in J. x indicates *P* < 0.05 from Torin1. DTT, dithiothreitol; ER, endoplasmic reticulum; FACS, fluorescence-activated cell sorting; mTORC1, mechanistic target of rapamycin complex 1; NRCM, neonatal rat ventricular cardiomyocyte; TM, tunicamycin.

We next examined the functional importance of mTORC1 activity for the ER stress response and cellular homeostasis. To this end, we blocked mTORC1 activation with the first-generation mTORC1 inhibitor, rapamycin (sirolimus), or the nonrapamycin-derivative mTOR inhibitor, Torin1, during ER stress induced by TM or DTT. Inhibition of mTORC1 resulted in attenuated upregulation of the protective ER-targeted proteins GRP78, GRP94, and PDIA6 in response to TM or DTT (Fig. 4, *A–D*). This may either reflect lower ER stress leading to reduced activation of the ER stress response, or it may instead stem from an involvement of mTORC1 in inducing these proteins during disturbed ER homeostasis. We therefore quantified UPR target gene expression as an indicator of ER stress. Transcript levels of Hsp90b1 (GRP94) and Hspa5 (GRP78) were significantly reduced with Torin1-mediated mTOR inhibition but not affected by rapamycin 3 h after TM treatment (Fig. 4*E*). No significant changes were observed in response to mTORC1 inhibition at 24 h (Fig. 4*F*). This suggests that mTOR inhibition may either directly attenuate ER stress or instead indicate involvement of mTOR in the transcriptional induction of the UPR during the early phase of ER stress. However, the observed impaired expression of the UPR at later time points appears to be due to an involvement of the mTORC1 pathway in the posttranscriptional regulation of the ER stress response.

To distinguish between the impact of acute and late mTORC1 activity on the upregulation of the UPR during ER stress, we attempted to inhibit mTORC1 exclusively during the early phase of ER stress, when mTORC1 activity is greatly increased. For this we cotreated NRCMs with TM and Torin1, followed by media removal after 1 to 6 h, washing cultures with media and adding new media, containing only TM. While reducing the time of Torin1 treatment was associated with increased expression of the UPR at 24 h, 1 h of Torin1 treatment was sufficient to inhibit mTORC1 for up to 24 h (Fig. 4*G*). Even reducing Torin1 incubation to 5 min resulted in sustained mTORC1 inhibition up to 24 h (data not shown), likely making this experimental setup less conclusive for studying the different functions of mTOR during the early and late phases of ER stress. Instead, we treated NRCMs for 12 h with TM, the time point at which we found mTORC1 activity is decreased to baseline levels (Fig. 1, *B* and *D*), followed by the addition of Torin1 to the culture media and quantifying GRP94, GRP78, and PDIA6 protein levels after an additional 12 h (Fig. 4*H*). This demonstrated that inhibition of mTORC1 exclusively during the late phase of ER stress was sufficient to reproduce the impaired upregulation of the UPR observed with continuous inhibition of mTORC1 for up to 24 h. In summary, this indicates that the attenuated expression of the UPR in Fig. 4, *A–D*, is at least partly based on an involvement of mTORC1 in posttranscriptional regulation of the UPR during the late phase of ER stress.

To further elucidate the functional relevance of mTORC1 activity during ER stress, we quantified cardiomyocyte survival in response to TM treatment and mTORC1 inhibition by rapamycin or Torin1. As expected, induction of ER stress by TM resulted in cell death by apoptosis (Fig. 4, *I–K*). Importantly, inhibition of mTORC1 with rapamycin or Torin1 resulted in a significant increase in cell death during ER stress compared with TM alone, as shown by cell counting or FACS analysis (Fig. 4, *J* and *K*), further highlighting the importance of the mTORC1 pathway as an adaptive response to ER stress.

Taken together, we conclude that mTORC1 undergoes biphasic regulation during ER stress, with activation in the early phase followed by inhibition and residual activity during chronic ER stress. Dynamic mTORC1 regulation can be mediated by ATF6 and is critical for an adaptive posttranscriptional response of cardiomyocytes to ER stress that controls cell survival.

## DISCUSSION

ER stress and protein misfolding are thought to be involved in the pathophysiology of various cardiac diseases (17). In this study, we describe a biphasic regulation of the mTORC1 pathway during ER stress in cardiomyocytes that have an important function in cellular adaptation to ER stress. Interestingly, mTORC1/S6 kinase was strongly activated within minutes of the onset of ER stress and inhibited only later, after several hours, if ER stress did not subside. Biphasic regulation of mTORC1/S6 kinase activity during ER stress can be mediated, at least in part, by ATF6 and pharmacological inhibition of mTORC1 increased cell death, apparently involving transcriptional and posttranscriptional regulation of expression of several adaptive UPR genes by mTORC1. Previously, ATF6 was shown to regulate mTORC1 activity by stimulating the expression of the small G protein Rheb (7, 9). However, activation of mTORC1/S6 kinase within minutes after disruption of ER protein homeostasis is likely to involve other mechanisms, such as direct binding of ATF6 to Rheb (9) or sensing of chaperone availability by mTORC1 (18). In addition, ER stress induces transient activation of PI3K and AKT (4), which lies upstream of mTORC1 and whose activity can be regulated by all three canonical branches of the ER stress response, IRE1, PERK, and ATF6, depending on cell type and stimulus (6, 19–21). Although ATF6 stimulated mTORC1/S6 kinase early during ER stress, ATF6 activation was also sufficient to inhibit mTORC1/S6 kinase when ER stress did not subside. Although the exact details of how this is mediated remain unknown, it may involve cross talk to other branches of the ER stress response, for example by PERK-dependent induction of the AKT inhibitor Trb3 (6). In conclusion, our results suggest that residual mTORC1 activity is essential for sufficient activation of the unfolded protein response and that its inhibition increases cell death during ER stress in cardiomyocytes.

## DATA AVAILABILITY

The data supporting this study are available upon request.

## GRANTS

C. H. acknowledges support by a Boehringer Ingelheim Fonds travel grant, the German Center for Cardiovascular Research (Deutsches Zentrum für Herz-Kreislaufforschung, DZHK) mobility program, and the German Heart Foundation (Deutsche Herzstiftung). H.A.K, N.F, M.V, and S.D. acknowledge DZHK Partner Site Heidelberg/Mannheim. S.D. acknowledges DZHK Excellence Program. M.V. acknowledges German Research Foundation (Deutsche Forschungsgemeinschaft) Grants DFG VO 1659 2/1, DFG VO 1659 2/2, DFG VO 1659 4/1, and DFG VO 1659 6/1; the Boehringer Ingelheim Foundation (Plus 3 Program); and the Baden-Württemberg Foundation (Baden-Württemberg Stiftung).

## DISCLOSURES

No conflicts of interest, financial or otherwise, are declared by the authors.

## AUTHOR CONTRIBUTIONS

C.H., M.V., and S.D. conceived and designed research; C.H., Z.L., and M.A. performed experiments; C.H., Z.L., and M.A. analyzed data; C.H. interpreted results of experiments; C.H. prepared figures; C.H. drafted manuscript; C.H., Z.L., M.A., R.J.K., H.A.K., N.F., C.C.G., M.V., and S.D. edited and revised manuscript; C.H., Z.L., M.A., R.J.K., H.A.K., N.F., C.C.G., M.V., and S.D. approved final version of manuscript.
